# Different modes of growth cone collapse in NG 108-15 cells

**DOI:** 10.1007/s00249-013-0907-z

**Published:** 2013-05-04

**Authors:** Philipp Rauch, Paul Heine, Barbara Goettgens, Josef A. Käs

**Affiliations:** 1 Institute of Experimental Physics I, University of Leipzig, Linnéstrasse 5, 04103 Leipzig, Germany; 2 Institute of Biology, University of Leipzig, Talstrasse 33, 04103 Leipzig, Germany

**Keywords:** Neuronal pathfinding, Cytoskeleton, Neurite retraction, Actin, Microtubules

## Abstract

In the fundamental process of neuronal path-finding, a growth cone at the tip of every neurite detects and follows multiple guidance cues regulating outgrowth and initiating directional changes. While the main focus of research lies on the cytoskeletal dynamics underlying growth cone advancement, we investigated collapse and retraction mechanisms in NG108-15 growth cones transiently transfected with mCherry-LifeAct and pCS2+/EMTB-3XGFP for filamentous actin and microtubules, respectively. Using fluorescence time lapse microscopy we could identify two distinct modes of growth cone collapse leading either to neurite retraction or to a controlled halt of neurite extension. In the latter case, lateral movement and folding of actin bundles (filopodia) confine microtubule extension and limit microtubule-based expansion processes without the necessity of a constantly engaged actin turnover machinery. We term this previously unreported second type *fold collapse* and suggest that it marks an intermediate-term mode of growth regulation closing the gap between full retraction and small scale fluctuations.

**Electronic supplementary material:**

The online version of this article (doi:10.1007/s00249-013-0907-z) contains supplementary material, which is available to authorized users.

## Introduction

Neuronal development during embryogenesis as well as regeneration after injury is a highly complex process that requires robust mechanisms on the single-cell level to produce reliable results. Therefore, a multitude of interacting and overlapping signaling and guidance mechanisms is necessary to regulate neuronal growth and steer neuronal processes towards their respective target areas.

**Fig. 1:**
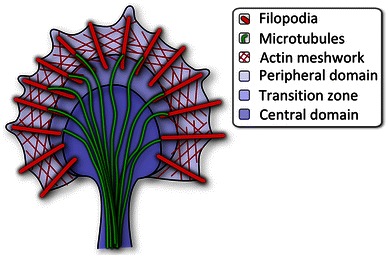
Growth Cone Structure The peripheral domain (*P-domain*) is a flat, often *fan-shaped* or *semi-circular *area where a dense filamentous actin (F-actin) meshwork is interspersed with bundles of actin filaments termed filopodia. At the distal edge actin polymerizes and thus pushes the edge forward and supports the retrograde, centripetal flow of actin. In the transition zone (*T-zone*) myosin motor proteins contract the actin network and contribute to the retrograde F-actin flow. Here F-actin is depolymerized into its globular monomeric form (G-actin) feeding the pool of free actin monomers available for transport to the leading edge and subsequent (re-)polymerization. The central domain (*C-domain*) is located at the end of the microtubule-filled neurite stump. Microtubules are tightly bundled in the neurite and splay apart within the *C-domain* of the growth cone. Individual MTs can reach out into the periphery and invade filopodia by aligning anti-parallel with and polymerizing against the retrograde actin flow

For this purpose, the highly complex and motile growth cone develops at the tip of outgrowing axons and, to a lesser extent, of dendrites.^1^ This hand-shaped entity contains mostly filaments of actin and microtubules (MTs) as dynamic and stabilizing structures. The typical structure of a growth cone is shown and described in Fig. 1. We also recommend the review by Lowery and Van Vactor (2009) for more detailed information. Growth cones can detect and process external stimuli and are able to respond sensitively to chemical guidance cues (Lockerbie 1987; Tessier-Lavigne and Goodman 1996; Dickson 2002; Mortimer et al. 2008). These positive or negative factors indirectly target cytoskeletal components and associated proteins triggering changes in the activity and distribution of actin structures and MTs which eventually lead to GC turning or retraction (Dent and Gertler 2003). The failure-free detection of the aforementioned cues is indispensable for the functioning of the growth cone and is one of the most important mechanisms underlying the generation of an intact nervous system. Thus, pruning of aberrant processes is at least as important for proper development, e.g. at the neuromuscular junction or the innervation of the eye as directional growth itself (Luo and O’Leary 2005). Consequently, alternating phases of active growth cone collapse or neurite retraction and outgrowth are integral features of neurite pathfinding (Kalil et al. 2000). On the one hand, many details are known about different modes of growth cone advancement and steering, especially concerning the interplay of actin polymerization and retrograde flow (Betz et al. 2009; Knorr et al. 2011) as well as actin-microtubule interactions (Zhou et al. 2002; Zhou and Cohan 2004). Collapse and retraction processes, on the other hand, are mostly evaluated in cases of partial or full retraction of the respective neurite. Short-term collapse and retraction, however, must not always conclude in complete truncation of the extension. Local retraction seems far more viable as a means of correcting possible detours. Halloran and colleagues were able to show that in living brain slices growth cone collapse, retraction, and pausing are not only commonplace, but also occur in a wide range of time scales from 2 to 15 min for 10–80 μm of retraction (Halloran and Kalil 1994). In the wake of these events a plethora of possible fates open up for the neurite, which include re-growth along neurite remnants (retraction fibers), outgrowth in a completely new direction, as well as a combination where the former neurite is kept as a branch. Inhibitory events in neurite path-finding leading to regular collapse and retraction play a decisive role even in neuronal regeneration, where growing neurites evade inhibitory substrates due to specific membrane bound signaling proteins (Patterson 1988).

On the scale of the growth cone edge, tightly regulated anti-parallel actin polymerization and retrograde flow enable fast switching from extension to retraction phases without inverting the whole machinery (Betz et al. 2009). While proteins from the myosin family contract the actin cytoskeleton, MT bound dynein family motor proteins can push from within the axonal shaft (Ahmad et al. 2000) with forces in the tens of piconewtons range (Rauch et al. 2013). There is convincing evidence that actin and MTs in combination with force generating motor proteins drive axonal advancement, retraction, and branching and are also crucial for reorientation of the growth cone after stimulation (Brandt 1998; Ahmad et al. 2000; Baas and Ahmad 2001; Andersen 2005; Kalil and Dent 2005). The contribution of peripheral actin polymerization to growth cone collapse remains elusive (Fan et al. 1993; Zhang et al. 2003; Gallo and Letourneau 2004). However, most studies investigating collapse mechanisms agree that an increase in actin-myosin contractility drives the retraction of the lamellipodium (Finnegan et al. 1992; Baas and Ahmad 2001; Zhang et al. 2003) and decreases the available space for MTs which are buckled and/or looped in the central domain (Tanaka 1991; Ertürk et al. 2007). Observations of retraction events after exposure to semaphorin 3A suggest that there are at least two independent processes during withdrawal: the collapse of the lamellipodium and the retraction of the neurite driven by different myosin subtypes (Gallo 2006). After application of lysophosphatidic acid (a Rho/Rho Kinase activator) Zhang et al. recorded substantial changes in actin cytoskeleton dynamics leading to a partial or full retraction of the neurite (Zhang et al. 2003). For large scale pathfinding of neuronal extensions, this might be a relevant mechanism. However, for the minute changes in position or orientation that may be required of a growth cone that is proximal to its target area, such considerable reorganization appears excessive.

It is plausible that an alternative process exists which collapses the growth cone without retracting the neurite and keeps dynein and microtubule pushing forces in check by inhibiting their extension outside the central domain. While in most processes related to growth cone turning and reorientation, a prominent role is ascribed to the dynamics of filopodia and their internal actin bundle structures, their function in GC collapse and retraction is largely unknown. Being among the most rigid structures in the growth cone renders them highly relevant to growth cone mechanics and a perfect target for signals triggering structural changes within the cytoskeleton [reviewed in (Mattila and Lappalainen 2008)].

In growth cones of NG108-15 neuroblastoma cells, which find application as model systems for neuronal signaling and growth processes [e.g. (Smalheiser 1991; Goshima et al. 1993; Tsuji et al. 2011)], we found evidence for an alternative, filopodia-based collapsing mechanism. It relies on local changes in filopodia dynamics and constitutes a mode of efficient mid-term inhibition of outgrowth not necessarily resulting in neurite retraction. We suggest that this newly found type of GC collapse closes the gap between the full withdrawal of a neuronal process into the cell body (*soma*) and the pausing of outgrowth achieved by actin retrograde flow and polymerization counterbalancing (Sato et al. 2011).

## Results

NG108-15 undergo neuron-characteristic, repeated cycles of neurite elongation and retraction (see supplemental movie S1) to explore different directions and evaluate regions of their environment most suitable for stable process formation. Under the influence of strong positive guidance cues this behavior is observed rather rarely while the noisy signaling environment of an unbiased dish culture (without adherent or diffusive signaling gradients) provokes frequent reorientation of outgrowing neurites. Our investigation of the retraction behavior of growth cones via analysis of laser scanning time lapse image series has revealed distinct behaviors occurring during GC collapse. These states are governed by different facets of filopodia, microtubule, and actin-myosin activity and their complex interactions within the dynamic GC cytoskeleton. All of the observed GC collapses are initiated by similar degradation of the lamellipodium. Nevertheless, they can be distinguished upon investigation of a few key attributes in their cytoskeletal dynamics and the overall morphology of the growth cone. Based on this, we were able to discern between two main types of collapse and retraction:

I. The complete collapse of the GC followed or accompanied by the full or partial retraction of the neurite. This was observed in 14 out of 25 collapsing GCs (56 %).
II. The transient collapse of peripheral GC structures (11 of 25 GCs, 44 %) that in some cases was followed by partial recovery of the system

In literature, mainly descriptions of type I retraction processes are found. These involve the complete disintegration of the GC, neurite retraction and the occasional re-growth of the process (Kapfhammer and Raper 1987; Patterson 1988; He et al. 2002; Wylie and Chantler 2003; Luo and O’Leary 2005; Obara et al. 2011). Our study mainly aims at characterizing the cytoskeletal activities occurring during the second, intermittent type of GC collapse. Based on this, we identify key processes distinguishing it from the full retraction case.

### Unlike complete retraction, transient (type II) GC collapse does not involve neurite retraction or C-domain area loss

**Fig. 2:**
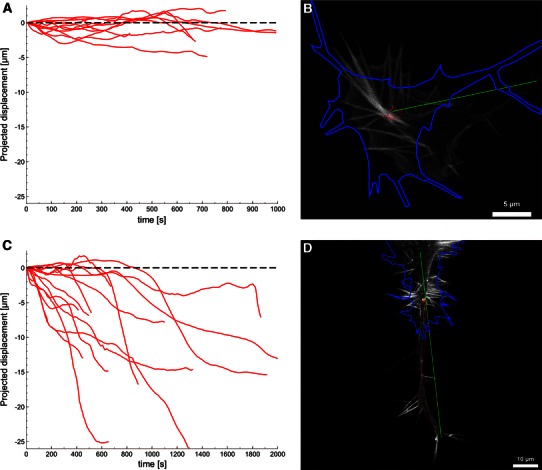
Growth cone displacement during fold collapse and pull retraction. *Graphs* **a** and **c** show the projected displacement of the GC from its initial position over time for fold collapse and pull retraction cases, respectively (zero displacement is indicated by a *dashed line*). Images **b** and **d** depict typical examples of fold collapse and pull retraction. The *grayscale* images show the first frame of the actin channel, while the *blue line* represents the final outline of the GC. The trace of GC movement is illustrated in *red*, while the *green line* represents the axis used to define the projected displacement. During fold collapse (**a**) growth cones generally do not retract a considerable distance, as can be seen in **b** by the trace which remains relatively close to the origin and the outline which shows no substantial GC movement during the recording. For pull retraction (**c**) large displacements towards the soma, such as portrayed in **d** (retraction distance is ~50 μm), are common. Examples for the tracing of a fold-collapsing and a retracting growth cone can be seen in movies S6 and S7, respectively

The most obvious characteristic of type II GC collapse is the persistent attachment of the GC’s central domain to the substrate. Throughout the whole process, central parts of the former GC remain intact and stationary while the periphery (i.e. the lamellipodium and filopodia) is disintegrated. We analyzed GC position during both variants of collapse and quantified their movement along the direction of outgrowth. The detection of the GC center was performed by calculating the center-of-mass (COM) from the GC outline (details of the method can be found in the Methods section). We found that type II GCs maintain their position as they collapse (see Fig. 2a). Small displacements (<5 μm) can be ascribed to changes in GC size and morphology. The loss of lamellipodial structures at the GC front typically leads to a relocation of the COM even though the C-domain does not move relative to the substrate. As shown in Fig. 2c, type I GCs on the other hand are retracted over substantial distances towards the cell soma or the nearest branching point of their neurite. The statistical significance of the differences in projected displacement for the two types was confirmed by application of a Student’s *t* test (*p* < 0.005). Retraction velocities during/after type I collapse strongly vary (corresponding to the curve slopes in Fig. 2c) and can reach values of more up to 9.6 μm/min (mean of maximal retraction speeds: 4.2 μm/min). These differences in GC displacement are the first indication that led us to believe that type II collapse is a more transient process than the full retraction case.

**Fig. 3:**
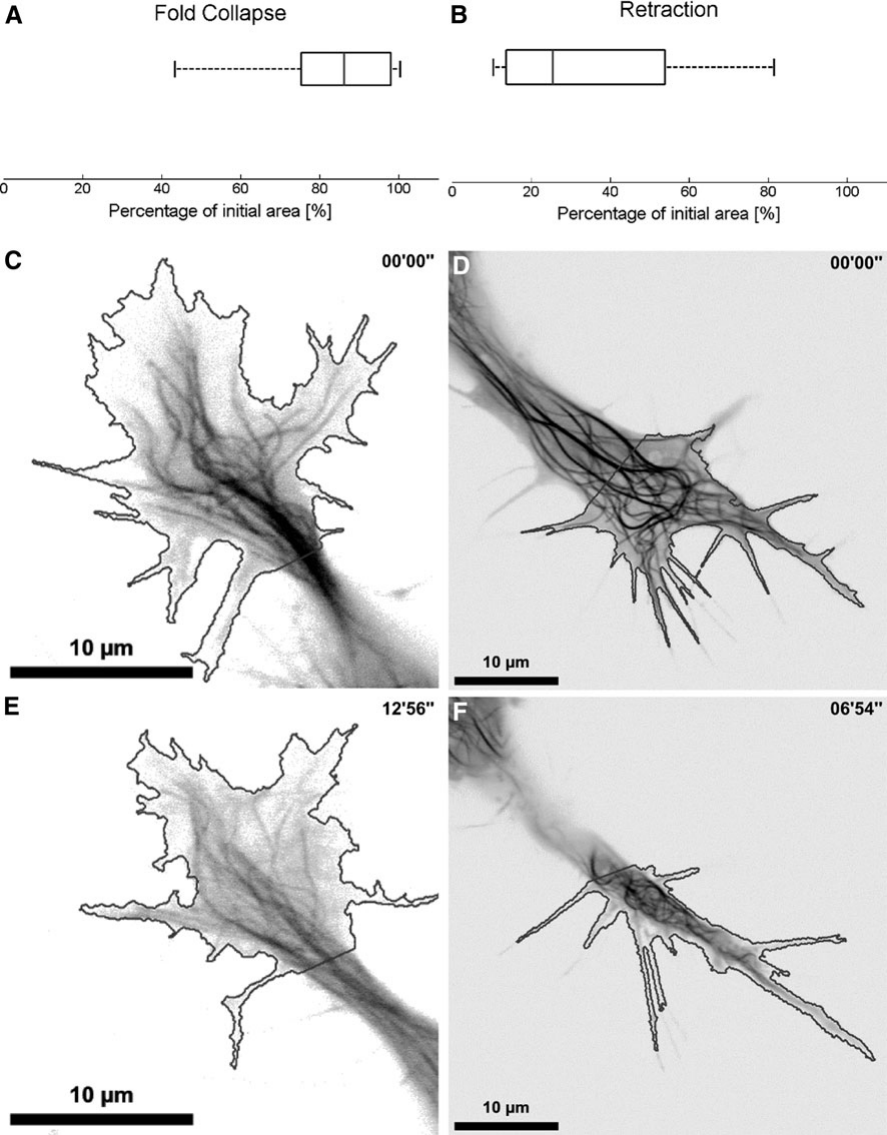
Reduction of growth cone area during fold collapse and pull retraction. GC area was evaluated using semi-automated edge detection algorithms before and after collapse. Histograms in **a** and **b** depict the reduction of growth cone area (as a percentage of the initial GC area) due to fold collapse and pull retraction, respectively. The mean values are 81.5 % (*n* = 9) and 34.5 % (*n* = 13) for fold and pull, respectively. The remaining frames show the MT channel before and after these events, the solid black outline reveals the GC area as derived from the F-actin channel. The example for fold collapse demonstrates that the GC area is only minimally reduced from image **c**–**e** and as a result MTs are only slightly compressed. In the case of pull retraction, however, growth cone width is substantially reduced (which can be seen in the change from **d** to **f**), which leads to a constriction of MTs formerly engaged in filopodia probing to the neurite axis and seems to increase MT buckling. Additional examples for fold collapse can be seen in movies M4 and M5, the effect of GC area collapse is shown in movie S2

The persistent stability of GC position indicates that substrate adhesions in the C-domain remain engaged during type II collapse while they necessarily disassemble during type I collapse to allow neurite retraction. Intact adhesions would not only stabilize the position of the neurite tip but also prevent the C-domain in type II GCs from collapsing. This assumption is supported in the temporal development of GC size as shown in Fig. 3. The comparison of GC area before and after collapse yields that type I GCs on average reduce their size by 65.5 % while type II GCs only lose 19.5 % of the initial area (*p* < 0.0001 Student’s *t* test).

Minor area reductions in type II cases can be attributed to the loss of peripheral actin structures that collapse onto the outline of the C-domain in a process that will be described in more detail below. The C-domain, however, almost completely maintains its size and shape. In the complete collapse cases, in contrast, where substrate contacts appear to be released, virtually all central and peripheral actin and microtubule structures concentrate in a very small area at the center of the former C-domain.

### GC area reduction constricts MT dynamics

**Fig. 4:**
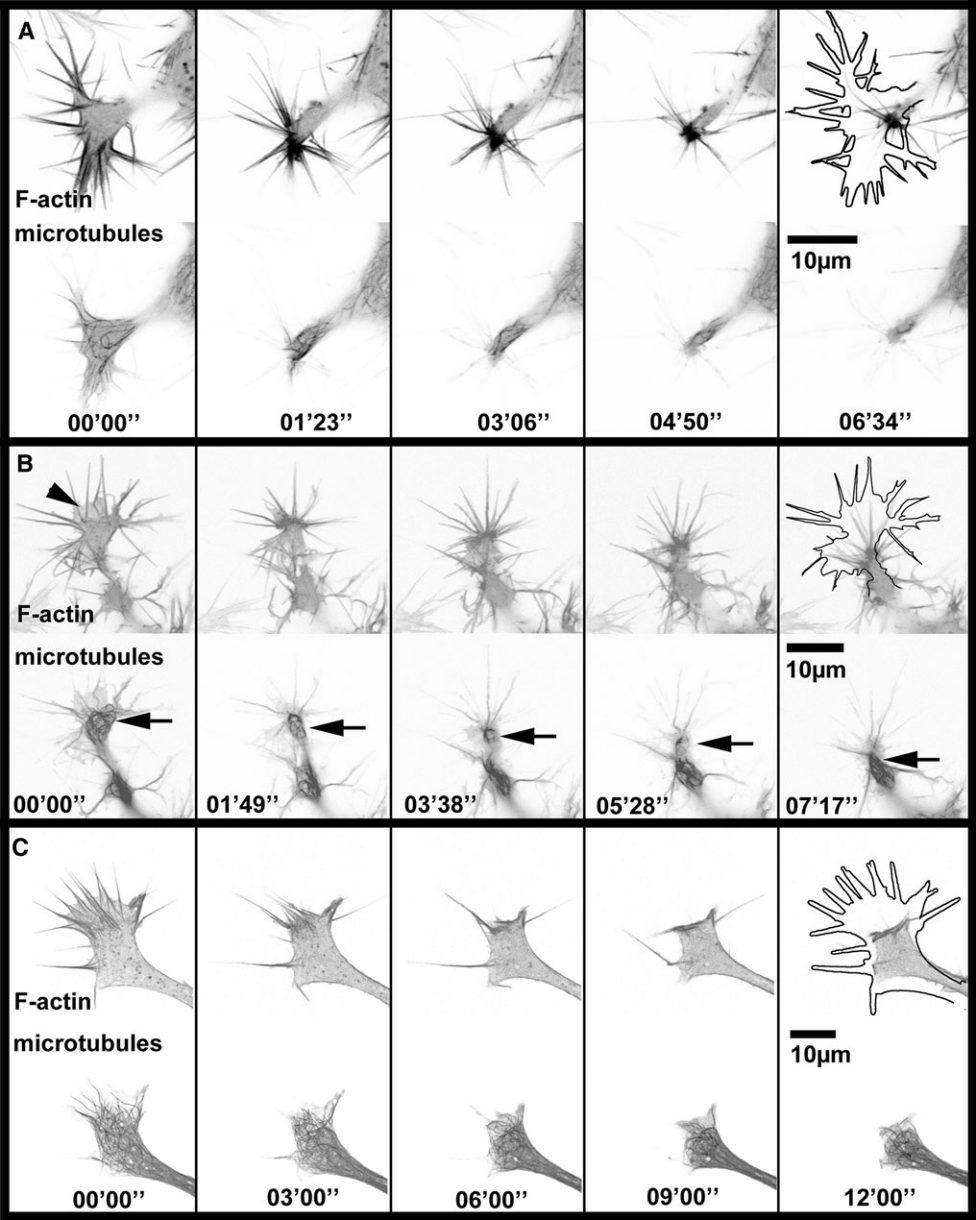
Different types of GC collapse in NG108-15. **a** Type I collapse with formation of a bulb-like structure (retraction bulb) prior to retraction. All structures with the exception of some filopodia are condensed in a region of the size of the neurite diameter. **b** Type I collapse and retraction with fibers. Filopodia (*top*) do not actively fold or significantly merge. Following a contractile motion of the *central area* and the neurite shaft, they are dragged behind, most likely due to incomplete detachment from the substrate. In the first image, remains of the lamellipodium can be seen (*arrowhead*). Microtubules initially spread in the GC are pulled back towards another MT rich area in the neurite (*arrow*). For the full length movie see supplemental material S2. **c** Type II collapse. Microtubules (*bottom row*) are pushed to the central area by the filopodia as they fold towards the -domain of the growth cone (*top row*). The process of folding is not accompanied by a retraction of the neurite shaft. For comparison, the *solid black lines* in the last panels represent the outline of the growth cone at time *t* = 00′00″. For the full length movie see supplemental material S4

The strong reduction in growth cone size during type I collapse and retraction naturally entails a pronounced effect on the configuration of MTs. They are pushed back centripetally through actin contraction (see movie S2 and Fig. 4a, b). MTs in the lamellipodium and the central domain are quickly condensed in the shrinking remnants of the former GC whereas filaments collocated with filopodia remain in a straight configuration until the filopodia themselves collapse. Eventually, the vast majority of microtubule filaments are concentrated in a bulb-like structure at the tip of the retracting neuron, entirely inhibiting their dynamics. Because of the strictly confined space they are unable to explore the periphery and, depending on the arrangement of the constricted growth cone, are aligned in bundles or buckle and form loops. This dense arrangement moves back towards the cell body as the neurite withdraws (arrow in Fig. 4b) and in case of full neurite retraction, merges into the soma’s cytoskeleton network.

During type II collapse we found turning filopodia to directly transport and bend associated MTs towards the GC center. At the same time frequent filopodia merging reduces the number of available tracks for MT outgrowth to the few remaining actin bundles. In addition, actin bundles from former filopodia fold down to the periphery of the remaining GC and form obstacles for MT polymerization at the circumference (see Figs. 4c, 6 and movies S4 and S5). The height of these obstacles is on the order of the GC height (~500 nm) since we could not observe three dimensional structures along the z-direction. All these processes are suitable to redirect tubulin polymerization forces away from the neurite extension path. Bent MTs simply polymerize into a different direction while in the case of caging, it is possible that tubulin polymerization pressure rather deforms MT filaments within the confined geometry than contributing to GC extension. The mechanism behind this resistance could originate from the proposed compressive forces that f-actin arcs apply in the transition zone (Geraldo and Gordon-Weeks 2009). Less pronounced shrinking of the central area in type II collapse results in condensed yet still dynamically entangled MTs that were never observed to traverse the aforementioned actin barriers. In previous experiments, we were able to show that individual MTs can generate forward directed pushing forces of about 30 pN (Rauch et al. 2013). The high density of dynamic MTs in collapsed GCs thus likely results in pressures against the actin frame in the range of 100 Pa. (25 MTs, each pushing with 30 pN confined by a semi-circular obstacle with *r* = 5 μm and wall height *h* = 500 nm).

### Transient collapse is characterized by reduction of filopodia number

In addition to GC displacement and area reduction, the dynamics of actin bundle filled filopodia turns out to be characteristic for the two types of GC collapse and retraction we observed. During type I collapse, filopodia remain stable and only show minor lateral mobility (see Fig. 4a, b). Actin bundles within filopodia persist even though the surrounding lamellipodium retracts. This leads to a spiky appearance of the former GC as retraction proceeds. Previous studies describe two different morphological variants that emerge after the lamellipodium has degraded: First, the formation of a so called *retraction bulb* when eventually all filopodia contract into a compact club-shaped structure. Alternatively, if filopodia remain attached to the substrate, the pulling out of so called *retraction fibers* was observed. These actin filled membrane tubes elongate as the neurite retracts until eventually their distal substrate contacts are released. Both processes (retraction bulb and fiber formation) have in common that contractile forces act on the collapsing growth cone and pull the neurite towards the cell body. Thus, both variants of type I collapse and retraction will be termed *pull retraction*. It was not always possible to distinguish clearly between the two subtypes of pull retraction. However, most GCs under investigation showed a tendency towards either the formation of a retraction bulb (Fig. 4a) or retraction fibers (Fig. 4b). Later, when we compare transient collapse (type II) and pull retraction (type I), we will no longer differentiate between the bulb and the fiber case.

The image series in Fig. 4c clearly shows that type II collapse is characterized by a sharp increase in filopodia merging and folding. Within 6 min, all actin bundles merge into three thick filopodia which subsequently kink and fold onto the outline of the GC’s C-domain. Because of this characteristic feature, we will call type II collapse *fold collapse*. The folding and rearrangement of previously radial actin bundles from filopodia leads to the formation of an actin-rich barrier at the circumference of the collapsing GC. As mentioned above, this confines dynamic MTs to the remaining area and hinders their advancement into the periphery. During pull retraction, in contrast, MTs follow the contracting actin system and move backward with the collapsed GC.

**Fig. 5:**
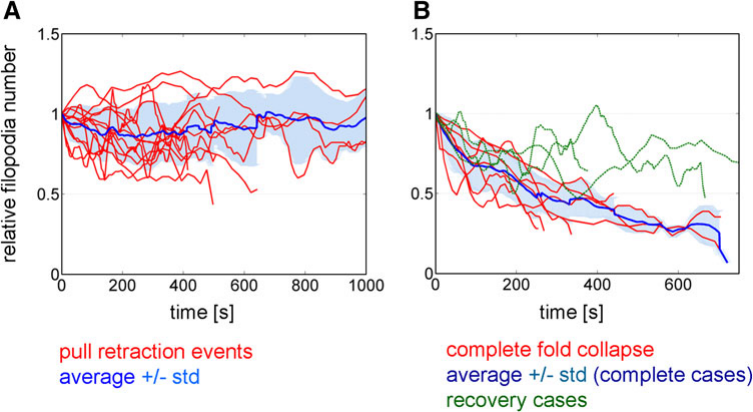
Reduction of filopodia number is characteristic for fold collapse. Filopodia were identified based on image intensity profiles recorded parallel to the outline of the C-domain. Initial filopodia numbers largely varied (7 ≤ *n*_0_ ≤ 24), thus all counts were normalized to the initial number *n*_0_. **a** During pull retraction filopodia numbers only undergo small fluctuations but on average (*n* = 13,* blue line*) show a tendency towards a constant number (an example is shown in movie S2). While some filopodia are pulled into the central domain, new formation hardly occurs. **b** In GCs undergoing fold collapse, the number of filopodia sharply decreases as soon as merging and folding commences. Red curves: Filopodia numbers of GCs undergoing complete fold collapse without significant recovery. Within 400 s, the filopodia number of all GCs drops to less than 50 % of the initial value. Green curves represent GCs which partially or temporarily recover after collapsing. Recovery includes the formation of new filopodia and a small lamellipodium as shown in movie S5. The* blue line* displays the average of all complete collapsing events without recovery (*n* = 7). In contrast to the pull retraction cases in **a**, a continuous decrease is observed

We evaluated filopodia numbers in collapsing GCs to characterize different retraction types by their temporal development (Fig. 5). During regular growth of spread-out NG108-15 growth cones on laminin coated surfaces the number and distribution of filopodia remained predominantly constant (data not shown, see supplemental movie S3). Filopodia merging is part of regular actin cytoskeleton dynamics and is usually compensated for through the formation of new actin bundles giving rise to small fluctuations around an average number of filopodia.

In the case of pull retraction, dynamics are suppressed in a sense that new filopodia rarely emerge while the existing actin bundles crumple or are pulled rearwards with the neurite. Fluctuations of the measured filopodia number partially result from filopodia temporarily being too close to each other, preventing them from being resolved individually and re-separating in the course of retraction. Within the accuracy of our method, the average number of filopodia remains unchanged throughout the entire retraction process (Fig. 5a).

A fold collapse event, in contrast, is characterized by a significant decrease in filopodia number. Figure 5b displays the filopodia numbers of GCs undergoing fold collapse (red curves). They show a characteristic drop at the onset of collapse corresponding to an increase in filopodia merging and folding. The recovery in three of the cases (green curves in Fig. 5b) can be ascribed to the reversible nature of this process: After an actin frame had developed through folding and merging, locally new filopodia and a limited lamellipodium re-emerged (exemplary GCs are shown in movies S4 and S5).

The statistical significance of these events was confirmed by evaluating the relative filopodia number at the end of the sequence of both event types by a Student’s *t* test (*p* < 0.0006).

**Fig. 6:**
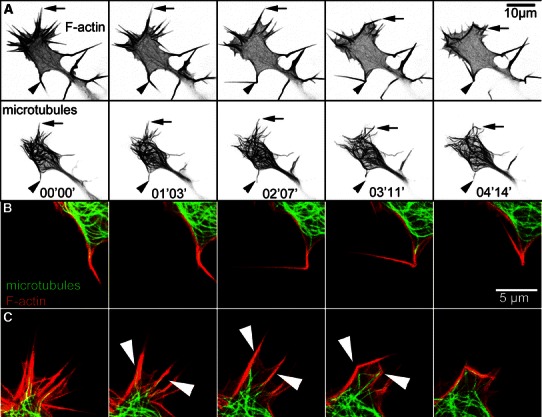
Folding of individual filopodia. **a** After lamellipodium degradation, filopodia (*top*) fold to the periphery of the GC and limit the available space for MT polymerization. The* black arrows* denote a pair of MTs (*bottom*) that invades filopodia and is transported inwards following the filopodium (*black arrows, top panel*) it is associated with. The *black arrowhead* denotes an MT targeted filopodium that folds up 180°. **b/c** Magnifications of the marked areas in **a**. Microtubules invade filopodia which fold and thus eliminate MT extension into the periphery. *Top row*: The filopodium bends into the z-direction and partially moves out of the imaging plane. Thus, it appears shorter in the first and second image. The kink develops at the tip of the invading microtubule. *Bottom row*: Two filopodia fold after they are targeted by microtubules (*white arrowheads*). After folding, MT fluctuations are confined by actin bundles. For the full fluorescence series see movie S5. The times displayed in the second row of a are valid for the respective column

During fold collapse we frequently observed the apparent breaking and kinking of actin bundles in filopodia lacking lamellipodial support. Figure 6a displays the collapse of a GC involving the folding of two large filopodia (black arrow and arrowhead). Magnifications of the respective regions can be found in Fig. 6b and c. The geometry of the folding actin bundles indicates that prior to folding, breaking or severing must have occurred. Otherwise, local contraction would rather lead to an arc-like deformation. The actin-microtubule overlay in Fig. 6b and c reveals that in this particular case, MTs had invaded the filopodia with their tips in close vicinity to the prospective breaking point. There is indeed evidence that MTs are able to influence filopodia mobility and increase the probability of merging (Schober et al. 2007). However, in most of the observed GCs, filopodia folding also occurred in the absence of MTs. Hence, an impact of MT positioning and actin bundle severing or folding cannot be stated conclusively based on our studies. Instead, we often observed filopodia that transport peripheral MTs towards the C-domain through folding and lateral motion.

## Discussion

### Different adhesion and contraction patterns result in different collapse and retraction types

**Fig. 7:**
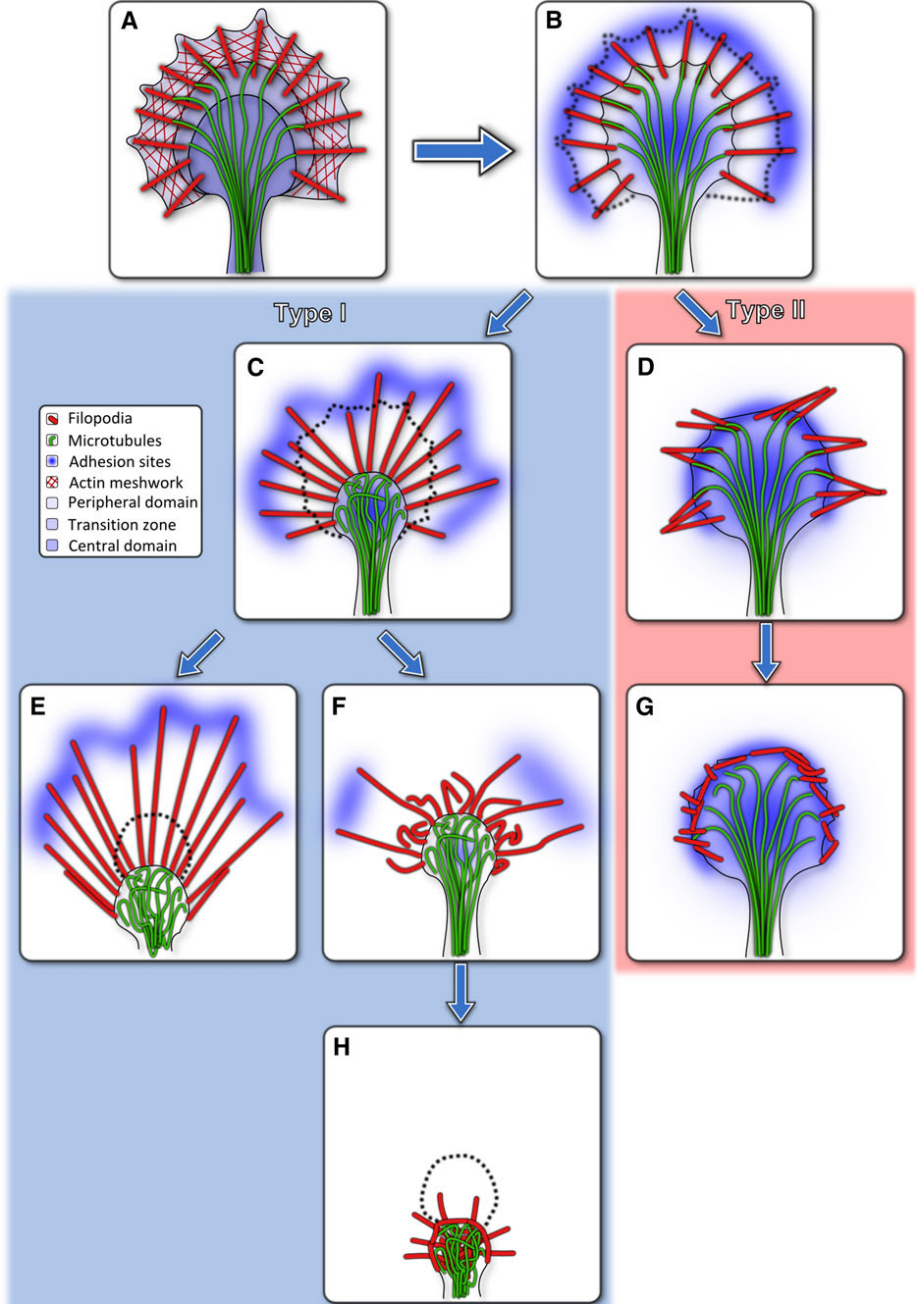
Schematic sequence of retraction dynamics. **a** Growth cone before collapse. The state of adhesion sites is not illustrated in box **a**; however, both peripheral filopodia and the central domain are assumed have intact adhesions. Designation of the three growth cone regions and the actin meshwork are omitted for clarity in the remaining boxes** b**–**h**.** b** Lamellipodium degradation: Common for all observed types of collapse is the initial lamellipodium retraction. **d, g** ***Fold collapse***: Filopodia form a dense peripheral actin structure. **c, e** ***Pull retraction*** (i) A subtype of pull retraction, that occurs when filopodia, instead of collapsing into the growth cone center, remain attached to the substrate is characterized by retraction fibers which the neurite drags behind. This variant of pull retraction leaves filopodia in a straight configuration. **c, f, h** Pull retraction (ii) Filopodia detach and are pulled back by contractile forces and collapse into themselves, while being dragged towards the center of the remaining growth cone. MTs appear intact while being confined within this highly condensed retraction bulb, consisting mainly of remnants of the former central domain. In all cases this type of collapse was accompanied by a retraction of the neurite tip which requires the detachment of the whole GC area from the substrate

We were able to discern aspects of cytoskeletal filament dynamics not only throughout the commonly described contractile retreat, which is accompanied by a partial or full retraction of the neurite, but also during formation of retraction bulbs and fibers. If we consider stationary parts of the GC substrate bound while those moving relative to the substrate are assumed to be detached, these observations indicate that both morphologies are essentially subtypes of conventional neurite retraction, which emerge due to variations in local substrate adhesion. Complete retraction is dominated by a collapse of the entire GC periphery into a small area within the central zone. When filopodia remain substrate bound during this collapse, the GC state can be sub-classified as retraction fiber retreat (Fig. 7a, b, c, e), whereas the caving-in of detached actin-bundles constitutes a retraction bulb (Fig. 7a, b, c, f, h).

More importantly, our studies specify another type of collapse involving the folding of filopodia towards the central area. Actin bundles originating in folding filopodia accumulate at the circumference of the former central domain and establish a two-dimensional cage-like superstructure. Remarkably, folding is not limited to systems with an intact lamellipodium and could be observed in fully-spread GCs as well as in such consisting merely of the central domain and the bare filopodia themselves. The lateral movement of filopodia embedded in lamellipodia was previously ascribed to interactions with the retrograde transport of the surrounding actin network. Depending on retrograde flow velocity and relative tilt angle, different rates of lateral motion were observed (Oldenbourg et al. 2000). In the case of free filopodia, however, the structures generating forces for lateral movement and folding are apparently localized within the actin bundles.

The most striking difference between pull retraction and fold collapse is that actin locally rearranges in a way that suggests the complete detachment of the periphery including filopodia while the C-domain remains stationary and fully spread. Unlike the pull retraction variant this enables a transient halt of the system while peripheral actin turnover is stopped. At the same time the GC is still able to rebuild locally advancing lamellipodium and filopodia structures which points to the fact that after an intermittent break, regular outgrowth can resume. A similar rearrangement of F-actin structures was described earlier (Torreano et al. 2005). However, in those studies the formation of a peripheral actin ring required the ML-7 (a myosin light chain kinase inhibitor) driven disassembly of actin bundles.

In motile cells and neuronal growth cones, myosin motors interacting with the actin cytoskeleton have been identified as the major source of contractile activity. They drive retrograde flow in the lamellipodium including filopodia (Lin et al. 1996) and are involved in mechanosensing, i.e. probing of the mechanical properties of the cellular environment (Mattila and Lappalainen 2008). The dynamics of the processes described herein originate in the movement of actin structures, which indicates that myosin motor activity is responsible for both the pulling retraction of a neurite, as well as the folding and lateral movement of filopodia in fold collapse. It appears to be the location of activity which is cause for the significant differences between the two processes. In pull retraction, contractile forces act in the central domain, the neurite shaft and along the actin bundles constituting filopodia. This leads to centripetal retraction of filopodia and a continuous retraction of the neurite dragging the GC behind. It was shown that upon according external stimuli, actin-myosin contraction in the T-zone and the neurite shaft is up-regulated inducing (pull) retraction (Zhang et al. 2003).

Previous studies showed that GC collapse and retraction can be separated into two independent processes (Gallo 2006; Brown et al. 2009): First, P-domain collapse, including a reduction in area, loss of the actin-based lamellipodium, and contraction of filopodia. Second, C-domain contraction and retraction of the neurite.

While the first mechanism seems to be myosin II-independent, the second requires activity of at least one isoform of myosin II. In contrast to pull retraction, the formation of new filopodia is not completely suppressed in the fold collapse case. In the periphery of the GC shown in Fig. 4a and movie S5, new small filopodia emerge while others fold towards the center. In addition, extension of new filopodia and local protrusion of the lamellipodium leading edge evidence that substrate contacts are not generally degraded during the process, but maintain the spatial stability of the whole system. We propose that the independent nature of C- and P-domain contraction in combination with either engaged or disengaged adhesion sites in the center and periphery of the GC leads to a number of possible outcomes for collapse/retraction events. This simplified classification covers all observed cases of collapse and retraction (see Fig. 7). However, it cannot address the question why during fold collapse lateral movement and folding of filopodia predominate, while in the case of bulb formation and complete retraction their movement is limited to the centripetal direction.

### Different mechanisms eliminate microtubule pushing

All collapse and retraction processes are based on the rearrangement of a previously forward directed kinetic system and an inversion of the net forces resulting from its internal dynamics. GC advancement is mainly driven by two mechanisms: (i) actin polymerization and contractile actin-myosin dynamics in the P-domain that result in forward movement through clutch-like substrate adhesions, and (ii) microtubule expansion through polymerization and MT-bound dyneins that push MTs forward from within the neurite shaft (Myers et al. 2006; Rauch et al. 2013). For both transient halt and long-range retraction, these two “motors” need to be disabled. Several actin-related mechanisms that occur during the retraction of the lamellipodium have been identified including the inactivation of radixin (a protein believed to link actin filaments to each other and the cell membrane) (Castelo and Jay 1999) and the activity of different myosin isoforms (Brown et al. 2009). However, as studies with drug-treated GCs show, actin inhibition alone does not prevent the elongation of neurites but only impairs their ability to respond to guidance signals (Marsh and Letourneau 1984; Bentley and Toroian-Raymond 1986). It was found that inhibiting MT dynamics is crucial for GC pausing (Hendricks and Jesuthasan 2009). Hence, in addition to the inhibition of actin polymerization and myosin activity, mechanisms are required to suppress pushing MTs. In the case of pull collapse and retraction, this seems to be achieved through the disassembly of central adhesion sites and an actin-myosin driven contraction of the whole system including the neurite shaft. During fold collapse, however, no significant contraction of the C-domain could be observed. It seems that the circumferential actin barrier built from folded filopodia assumes the function of limiting MT-based GC advancement. Microtubules remain dynamic but confined to the former C-domain, able to resume their pushing function as soon as outgrowth continues. Hence, new filopodia and lamellipodium structures that emerge during partial recovery are initially void of MTs. Many studies confirmed the crucial role of MTs for GC advancement, steering and neurite branching (Brandt 1998; Dent and Kalil 2001; Zhou and Cohan 2004; Ertürk et al. 2007) which suggests that a lack of this cytoskeletal component in the peripheral domain will impair the ability of the GC to branch or turn. Along with the persistent spreading of the C-domain this reversible regulation of MT pushing indicates that fold collapse represents a transient mid-term pausing of neurite extension.

### Possible mechanisms underlying filopodia folding

Centripetal contractility at the aforementioned locations, however, is insufficient to explain the extreme lateral movement and sharp kinks in filopodia which we observed during fold collapse. Folded filopodia are apparently disintegrated at a single point and thus separated into linked segments which retain their straight configuration throughout the process. This indicates highly localized mechanisms regulating actin-myosin activity and the destabilization of actin bundle structures. We can only speculate about the mechanisms leading to the strictly confined weakening of actin bundles and their local folding without visible bending of the remaining segments. Possible players in this context might be myosin minifilaments that were previously detected in the contractile stress fibers of fibroblasts (Svitkina et al. 1989) and, more recently, in neuronal growth cones (Bridgman 2002). Studies comparing the motility of myosin minifilaments to that of purified motor domains confirm that motor activity in the filamentous form results in contraction rather than forward movement (Kolega 2006).

**Fig. 8:**
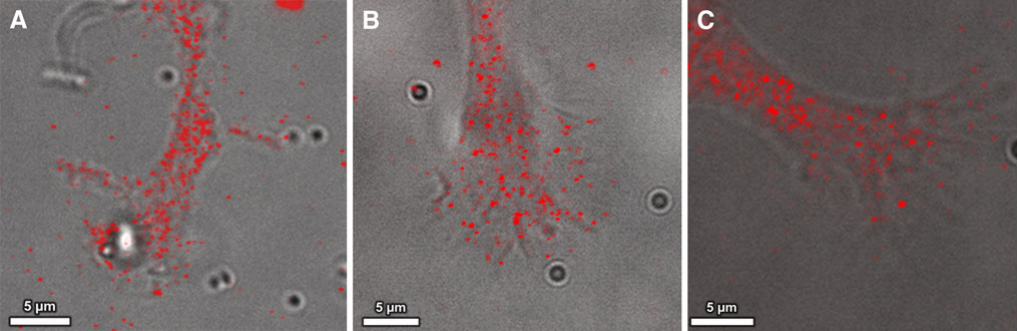
Gelsolin is localized in NG108-15 growth cones. Bright field microscopy images are overlaid with immunostains of gelsolin antibodies (*red*) to visualize the distribution of gelsolin in three representative NG108-15 growth cones. Gelsolin clusters are distributed all over the growth cone and also co-localize with filopodia. The presence of high intensity clusters in the vicinity of filopodial actin bundles supports the suggested role of gelsolin in filopodia dissociation and folding

The occurrence of sharp kinks in folding filopodia also requires local defects in the underlying rigid actin bundles. As mentioned above, MTs and associated proteins cannot be held responsible for actin bundle severing. This raises the question, which molecular mechanism may induce filopodia breaking prior to folding? Among the few proteins known to cut actin filaments, *gelsolin* seems to be the most promising candidate for two reasons: First of all, the presence of gelsolin in growth cones and filopodia of primary neurons and neuronal cell lines was shown with immunostaining techniques (Tanaka et al. 1993); second of all, neurons extracted from mice lacking gelsolin (gelsolin-null mice) show abnormal filopodia dynamics and an impaired ability to retract GC filopodia (Lu et al. 1997). Since the expression of gelsolin in the growth cones of NG108-15 cells was not previously reported, we performed immunostaining of fixed cells with gelsolin antibodies. Figure 8 displays laser scanning and bright field images of the stained cells and clearly shows the presence of gelsolin in NG108-15 growth cones. Gelsolin is not homogeneously distributed but clusters in high density punctae which can also be found in the GC periphery close to filopodia. Selective activation of these point-like gelsolin accumulations might be responsible for local actin bundle dissection and create prospective sites for kinks in folding filopodia.

Based on this, we suggest that in type II collapse, gelsolin is activated locally and weakens actin bundles within filopodia prior to folding. Clustered myosin motor proteins or myosin minifilaments drive the contraction of the cut bundles and cause their folding at the points of minimum stability resulting in sharp kinks rather than globally deformed bundles. The mechanisms triggering the local destabilization of actin bundles in type II but not in type I collapse, however, remain to be investigated.

### Concluding remark

Here we report on a previously undocumented type of GC collapse characterized by lamellipodium degradation, continuous C-domain adhesion and, most remarkably, the pronounced folding of filopodial actin bundles that mechanically limit MT driven GC advancement. Considering that within a physiological environment guidance cues are subtle and may overlap, it seems plausible that full retraction is not necessary in all situations. Especially repellant signals usually do not aim at a complete retraction of the neurite but are rather present where outgrowth is supposed to halt. Under these conditions the transient *fold collapse* we characterize here excellently fits the requirements of a mid-term halt without losing overall integrity and stability. Nevertheless, the availability of a mechanism for fast and complete retraction is crucial. It allows the neuron to quickly withdraw one or all of its processes to avoid further damage, e.g. after mechanical over-stimulation or under the influence of highly repellent or overdosed artificially applied guidance cues. We were able to show that the cytoskeletal components present in neuronal GCs can accomplish not only complete retraction but also a to date uncharacterized transient type of collapse, most likely driven by local variations in contractility and adhesion patterns.

## Materials and methods

### Cell culture and transfection

NG108-15 neuroblastoma cells were purchased from ATCC (Manassas, VA, USA) and cultured in standard growth medium composed of Dulbecco’s Modified Eagle Medium (DMEM) supplemented with 10 % fetal calf serum and 1 % penicillin/streptomycin solution (all purchased from PAA, Pasching, Austria). Cells were transiently co-transfected with mCherry-LifeAct plasmids (IBIDI, Martinsried, Germany) for F-actin visualization and pCS2+/EMTB-3XGFP plasmids (kindly provided by the group of Ewa Paluch, Max Planck Institute of Molecular Cell Biology and Genetics, Dresden, Germany) for microtubule visualization. Transfections were performed 24-72 h prior to image acquisition with liposome-based Metafectene^®^Easy (Biontex, Martinsried, Germany) according to their standard protocol.

The NG108-15 hybrid cell line exhibits certain characteristic features of nerve cells, such as differentiation and the spurting of neurite-like processes, which are known to form synapses that are functional on the presynaptic side (Hamprecht, B., 1977). We chose this cell line since these cells readily respond to transfection treatments and their well-pronounced growth cones constitute ideal model systems for investigations of the underlying cytoskeleton.

### Image acquisition

For image acquisition, cells were seeded on custom-made glass bottom petri dishes or μ-slide 18-wells (IBIDI, Martinsried, Germany) and supplied with phenol-red free Leibovitz’s L-15 medium with 2 % B-27^®^ supplement (both from Invitrogen, Darmstadt, Germany). All images were captured on a Leica TCS SP5 confocal laser scanning microscope equipped with an HCX PL APO CS 63.0 × 1.40 oil immersion objective (Leica Microsystems, Wetzlar, Germany). For better visualization, images in Figs. 2, 4 and 5 were contrast enhanced and inverted using image processing software.

### Image analysis

All image analysis was performed using custom written MATLAB scripts (The MathWorks,Inc., Natick, MA, USA).

Filopodia numbers of growth cones displayed in Fig. 3: A manually drawn line crossing all filopodia in the raw image is the basis of further analysis steps. The intensity profile along this line is evaluated and peak intensities above a critical threshold are identified as filopodia. Thresholds need to be adapted to each image series individually, accounting for initial fluorescence intensity and bleaching during image acquisition.

Growth cone area and outlines, as shown in Fig. 5: An initial freehand selection of the growth cone at the beginning and the conclusion of pull retraction or fold collapse is the basis for the threshold-based area detection algorithm. In detail, the complete growth cone is coarsely selected to crop the region of interest (ROI) from the full-size fluorescence image and to define the cut-off line at the growth cone’s neck. Within this region of interest, a threshold-based detection of the GC outline is performed.

Growth cone positions in Fig. 6: After pre-selection of the approximate growth cone region (region of interest, ROI) in the first image of a series, an intensity-weighted center of mass (COM) is determined. By this, rearrangements of the actin cytoskeleton (and thus a redistribution of fluorescence intensity) as well as shape changes of the growth cone contribute to the calculation of the COM position. This COM is set as the initial position for tracing. The subsequent image is analyzed based on the COM-centered ROI from the previous frame and a new growth cone position is determined. To calculate the projected displacement along the axis of neurite outgrowth, a line is manually drawn with a fixed point at the origin (first COM) and pointed towards the neurite base. This constitutes the y-axis of a translated and rotated coordinate system, with its origin in the COM of the first frame. GC displacement towards the soma then merely corresponds to the y-coordinate of the COM. A characteristic example for the growth cone position analysis of both fold collapse and pull retraction can be viewed in Fig. 2 and supplemental movies S6 and S7, respectively.

### Gelsolin immunostaining

For gelsolin immunostains we used a Cy3 conjugated rabbit anti-gelsolin polyclonal antibody (purchased from Gentaur Molecular Products, Germany) and a Goat anti-Rabbit IgG (H + L)-Cy3 secondary antibody (Dianova, Germany). Immunocytochemistry was performed according to standard protocols employing 4 % Paraformaldehyde as a fixation solution, 0.1 % Triton X-100 as a permeabilization solution, 1 % BSA in 1xPBS as a blocking solution, and 0.05 % Tween-20 as a wash buffer. Cells were washed with PBS Buffer (at 37 °C). Cells were then fixed with Paraformaldehyde for 30 min (at room temperature) and subsequently washed two times with wash buffer. Triton X-100 was applied for 3 min to permeabilize the cells after which the cells were washed two times with wash buffer. The BSA blocking solution was applied for 30 min (at room temperature). Next, 5 μL Cy3-anti-gelsolin were added into 750 μL blocking solution for 60 min (at room temperature) afterwards cells were washed two times with wash buffer. Then 5 μL Cy3-Goat-Anti-Rabbit were added into 750 μL blocking solution for 60 min (at room temperature) after which cells were washed three times with wash buffer and covered with PBS.

## Electronic supplementary material

Below is the link to the electronic supplementary material.

Supplementary material 1 (DOC 27 kb)

Supplementary material 2 (AVI 2573 kb)

Supplementary material 3 (AVI 2992 kb)

Supplementary material 4 (AVI 2027 kb)

Supplementary material 5 (AVI 2409 kb)

Supplementary material 6 (AVI 3896 kb)

Supplementary material 7 (AVI 1967 kb)

Supplementary material 8 (AVI 2470 kb)

## Abbreviations

GC: Growth cone
MT: Microtubule
P-domain: Peripheral domain (of the growth cone)
C-domain: Central domain (of the growth cone)
T-zone: Transition zone (of the growth cone)
F-actin: Filamentous actin
G-actin: Globular (monomeric) Actin
ATP: Adenosine triphosphate
GFP: Green fluorescent protein

## Footnotes

1 In the following, we will use the term neurites to include both axons and dendrites, since discerning axons from dendrites is not always possible in in vitro cultures.
