# Supplementary material for: Different modes of growth cone collapse in NG 108-15 cells
Source: Eur Biophys J. 2013 May 4;42(8):591–605. doi: 10.1007/s00249-013-0907-z (PMC3705140; doi:10.1007/s00249-013-0907-z)
Supplement: Supplementary file 1 — Supplementary material 1 (DOC 27 kb) [file 249_2013_907_MOESM1_ESM.doc]

**Supplemental Movie Legends**

Movie S1: Phase contrast movie of an NG108-15 neuro-blastoma cell on laminin coated glass. Neurites continuously grow, pause and retract on various time and length scales.

Movie S2, related to figure 2B: Fluorescence movie (green: microtubules, red: F-actin) of a retracting NG108-15 growth cone. After lamellipodium degradation, filopodia dynamics are suppressed. All cytoskeletal structures are compressed and pulled towards the soma.

Movie S3: Fluorescence movie (green: microtubules, red: F-actin) of an outgrowing NG108-15 growth cone. The number of filopodia remains constant since merging is compensated by the formation of new actin bundles.

Movie S4, related to figure 2 and 5: Fluorescence movie (green: microtubules, red: F-actin) of an NG108-15 growth cone undergoing fold collapse. Filopodia merge and fold towards the central domain. Microtubules are condensed in the central area and form loops. During collapse, the GC remains attached and does not retract.

Movie S5, related to figure 3 and 4: Fluorescence movie (green: microtubules, red: F-actin) of an NG108-15 growth cone undergoing fold collapse with partial recovery. After most filopodia have collapsed, a small lamellipodium with filopodia locally emerges at the periphery.

Movie S6, related to figure 6: Tracing the position of a collapsing GC using the center-of-mass algorithm. Red dots indicate the calculated GC positions. During folding, the central domain remains stationary.

Movie S7, related to figure 6: Tracing the position of a retracting GC using the center-of-mass algorithm. Red dots indicate the calculated GC positions. The neurite fully retracts over a distance of approximately 25 µm.
